# Project LifeSkills - a randomized controlled efficacy trial of a culturally tailored, empowerment-based, and group-delivered HIV prevention intervention for young transgender women: study protocol

**DOI:** 10.1186/s12889-017-4734-5

**Published:** 2017-09-16

**Authors:** Lisa M. Kuhns, Matthew J. Mimiaga, Sari L. Reisner, Katie Biello, Robert Garofalo

**Affiliations:** 1 Division of Adolescent Medicine, Ann & Robert H. Lurie Children’s Hospital, 225 E. Chicago Avenue, Box 161, Chicago, IL 60611 USA; 2 Department of Pediatrics, Northwestern University, Feinberg School of Medicine, Chicago, IL USA; 3 The Fenway Institute, Fenway Health, Boston, MA USA; 4 Departments of Behavioral & Social Health Sciences and Epidemiology, Brown University School of Public Health, Providence, RI USA; 5 Center for Health Equity Research, Brown University, Providence, RI USA; 6 Department of Psychiatry & Human Behavior, Alpert Medical School, Brown University, Providence, RI USA; 7 Division of General Pediatrics, Boston Children’s Hospital/Harvard Medical School, Boston, MA USA; 8 Department of Epidemiology, Harvard T.H. Chan School of Public Health, Boston, MA USA

**Keywords:** HIV prevention, Transgender women, Intervention

## Abstract

**Background:**

Transgender women in the U.S. have an alarmingly high incidence rate of HIV infection; condomless anal and vaginal sex is the primary risk behavior driving transmission. Young transgender women are the subpopulation at the highest risk for HIV. Despite this, there are no published randomized controlled efficacy trials testing interventions to reduce sexual risk for HIV among this group. This paper describes the design of a group-based intervention trial to reduce sexual risk for HIV acquisition and transmission in young transgender women.

**Methods:**

This study, funded by the National Institutes of Health, is a randomized controlled trial of a culturally-specific, empowerment-based, and group-delivered six-session HIV prevention intervention, Project LifeSkills, among sexually active young transgender women, ages 16-29 years in Boston and Chicago. Participants are randomized (2:2:1) to either the LifeSkills intervention, standard of care only, or a diet and nutrition time- and attention-matched control. At enrollment, all participants receive standardized HIV pre- and post-test counseling and screening for HIV and urogenital gonorrhea and chlamydia infections. The primary outcome is difference in the rate of change in the number of self-reported condomless anal or vaginal sex acts during the prior 4-months, assessed at baseline, 4-, 8-, and 12-month follow-up visits.

**Discussion:**

Behavioral interventions to reduce sexual risk for HIV acquisition and transmission are sorely needed for young transgender women. This study will provide evidence to determine feasibility and efficacy in one of the first rigorously designed trials for this population.

**Trial registration:**

ClinicalTrials.gov number, NCT01575938, registered March 29, 2012.

## Background

Available evidence suggests that the prevalence of HIV infection is disproportionately high among transgender women, including young transgender women (YTW) both in the U.S. and globally. A meta-analysis of the global burden of HIV infection in transgender women found an HIV prevalence of 19.1% (95% CI = 17.4-20.7) and a 49-fold increased odds of HIV infection compared with all adults of reproductive age [1]. In the U.S., a meta-analysis of 29 studies [2] found a prevalence of 27.7% laboratory-confirmed HIV infection (four studies) and 11.8% self-reported (18 studies) among transgender women. Furthermore, data from local testing of over 500 transgender women (with no known prior positive HIV test results) in Miami, San Francisco, and Los Angeles found the highest number of new infections among those ages 20-29 years (i.e., 45% of all cases) [3], suggesting disproportionate HIV incidence among younger transgender women in particular.

Condomless sex, particularly anal and vaginal sex, represents transgender women’s primary risk for HIV acquisition and transmission with high rates of sexual risk reported in small studies, including among YTW [2, 4, 5]. Recent findings suggest that inconsistent condom use during anal sex with HIV-infected partners offers little to no protection against HIV infection, and that consistent condom use over time is rare [6]. While biomedical interventions, including pre-exposure prophylaxis (PrEP), have demonstrated safety and efficacy to prevent HIV infection among men who have sex with men and transgender women in clinical trials [7–9], analysis of uptake of PrEP in practice has been limited among transgender women (<10%) [10]. Furthermore, among transgender women taking PrEP in the iPrex trial, evidence suggests medication adherence was suboptimal [11]. Given challenges with both uptake and adherence to PrEP, combination HIV prevention approaches, including promotion of condom use, are more effective than PrEP alone [12] in high risk populations and are recommended by the Centers for Disease Control and Prevention (CDC) [13].

Only a small number of (non-randomized) interventions have attempted to reduce sexual risk in transgender women. In a critical review of behavioral interventions for HIV prevention among transgender women undertaken by members of our research team [14], we identified only five interventions formally tested for transgender women (one for YTW, in particular). Four of 5 studies documented small to moderate effects with evidence of diminishing effects over time [14]. None of these studies have been of sufficient quality to be included in the CDC’s compendium of HIV prevention interventions (https://www.cdc.gov/hiv/research/interventionresearch/compendium/).

### Study objectives

The primary objective of this study is to test the efficacy of the LifeSkills intervention to reduce sexual risk for HIV acquisition and transmission among young transgender women, ages 16-29 years. A secondary objective is to test hypothesized social, cognitive and behavioral mediators of the intervention effect.

## Methods/design

### Design

**Fig. 1:**
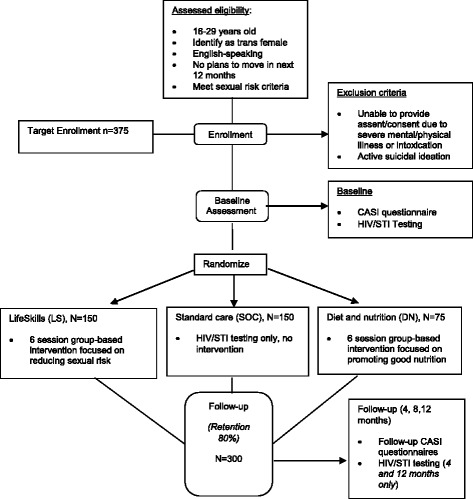
Flow chart of study intervention process

This study is a three-arm randomized controlled trial targeting young transgender women, ages 16-29 years who are at high risk of HIV acquisition or transmission due to sexual risk behavior, conducted in two cities in the U.S.: Boston and Chicago. Participants are randomized to receive the LifeSkills group-based intervention, standard-of-care preventive care only, or a time- and attention-matched group-based control intervention (non-active). All study groups receive repeat standard preventive care which consists of HIV testing with pre-posttest counseling and urogenital screening for gonorrhea and Chlamydia. Participants who are randomized are followed for 12 months with visits conducted at 4-month intervals: 4, 8, and 12-month post-randomization (Fig. 1).

### Identification and recruitment of participants

Participants are recruited via outreach to community-based organizations and events, public spaces (e.g., bars and clubs), and social media outreach and advertisement (e.g., Facebook, Craig’s List); and through direct outreach by peer recruiters and transgender community leaders. Grounded in community-based participatory research principles [15], recruitment is carried out with input from local transgender communities in each city. Eligibility criteria include: (1) ages 16-29 years; (2) assigned male sex at birth and, at the time of enrollment, self-identify as a woman, female, transgender woman, transfemale, male-to-female (MTF), or on the trans feminine spectrum; (3) English-speaking; (4) no plans to move from the local area during the 12-month study period; and (5) self-reported sexual risk in the previous 4 months (i.e., condomless anal or vaginal intercourse; anal or vaginal intercourse with more than one sexual partner; anal or vaginal sex in exchange of money, food, shelter; or diagnosis with HIV or another sexually transmitted infection). Participants are excluded if they are unable to provide assent/consent due to severe mental/physical illness or intoxication; or if they are actively suicidal at the time of enrollment. Potential participants may enroll when these conditions resolve.

### Randomization

On a rolling basis, cohorts are assembled for a randomization visit (*n* = 10-15) in which individuals are randomized in blocks of 5 (2:2:1) to either LifeSkills (LS), standard of care (SOC) or diet and nutrition (DN) attention control. The random assignments are generated by computerized random number generator. Random assignments are concealed from both participants and study staff until they are revealed at the randomization visit, after each complete cohort has been assembled.

### Description of the intervention: Project LifeSkills

The LifeSkills intervention was developed to address the specific structural, developmental, and interpersonal challenges to sexual safety based upon the well-documented social realities of YTW [16, 17] and demonstrated feasibility and initial efficacy in a CDC-funded pilot trial [16]. Theorized mediators of the intervention effect include empowerment processes (e.g., increases in collective self-esteem and integration) as well as traditional HIV related prevention and health promotion targets (i.e., HIV-related knowledge, motivation, and behavioral skills). Empowerment has been described as a social process in which individuals (and communities) gain mastery over their lives [18]; it typically involves the development of skills, knowledge, and confidence which allows them to overcome obstacles in their personal and communal lives [18–21]. Empowerment processes may facilitate behavior change, building on the resiliencies, strengths, and capabilities of YTW as a collective community [22, 23], and help to buffer the effects of social and economic marginalization. There is mounting evidence that social and economic factors are associated with high rates of HIV transmission and adverse HIV-related outcomes in high risk groups [24]. The Information, Motivation, Behavioral Skills (IMB) model integrates theory and research from both health and social psychology literatures and has been applied to a wide array of wellness-promotion and health behaviors [25, 26]. With respect to HIV prevention, IMB focuses on the set of informational, motivational, and behavioral skills that are conceptually and empirically associated with safer sex. The Life Skills intervention targets information, motivation, and behavioral components consistent with the IMB model for HIV sexual risk reduction in YTW.

The LifeSkills curriculum is manualized and includes six sessions (Table 1): 1: Pride, 2: communication and respect; 3: skills building, 4: knowledge and self-protection, 5: partner negotiation, 6: tying it all together) to communicate basic HIV-related information (e.g., transmission modes and related risks), develop motivation (e.g., to protect oneself), and promote behavioral skill development (e.g., condom use, sexual partner communication and negotiation) through an empowerment-based approach [17]. The Life Skills curriculum incorporates interactive activities, in-depth discussions, videos, games, and role-plays to help participants develop, practice, and integrate new skills into “real-life” situations. Integrated throughout the curriculum is information on gender transition/affirmation and experiences and scenarios specific to transgender women’s experiences. Each session begins by asking participants to share one proud moment that occurred in the previous week to focus on the positive aspects of their lives and set the tone for the intervention session. Each session ends with a ceremony in which participants are given a small gift (i.e., key chain, mirror) and asked to describe one thing they learned from the day’s session that they could teach other young women, which serves to reinforce key intervention messages. During the sixth session, participants develop a personal risk reduction plan designed to identify risk behavior and alternative behavior options. The intervention is delivered by two trained transgender women in 2-h small-group sessions twice a week for three consecutive weeks. Facilitators are trained using a standard protocol. To monitor intervention fidelity, intervention sessions are audio-recorded, reviewed, and rated by an outside evaluator. Study facilitators also complete self-rating forms. Feedback is provided in weekly supervision sessions by the study coordinator at each site based on review of rating forms.

**Table 1:** Summary of LifeSkills theoretical constructs and session topics

Session	Title	Theoretical Constructs:
1	Pride	Transgender adaptation/ integration; collective self-esteem/empowerment
2	Communication and Respect	Information (HIV knowledge), skills building for communication
3	Skills Building – Housing, Employment, Medical Services	Building skills to meet basic needs and applying communication skills
4	Knowledge and Self-Protection	Information, Motivation (attitudes, norms, and intentions for safer sex), basic skill building for condom use.
5	Partner Negotiation	Behavioral skills (discussing sex and condom use with sexual partners), sexual contexts, drug/alcohol use
6	Tying it all Together	All Constructs Reviewed

### Time- and attention-matched control: Diet and nutrition

The time and attention-matched control intervention is also manualized and delivered at the same time/place as the LifeSkills intervention in six sessions and facilitated by a transgender woman. Control manual content was drawn from recommendations of the United States Department of Agriculture (USDA; https://www.choosemyplate.gov). The control intervention sessions include: an introductory session, followed by five sessions devoted to the major food groups. Control group sessions include interactive activities and games and are of similar length as the LS sessions.

### Standard-of-care prevention services

Participants in all study conditions receive pre-posttest counseling coupled with HIV screening (i.e., third generation testing algorithms at each site for those reporting HIV-negative or unknown status) per the RESPECT-2 protocol [27] and urogenital gonorrhea and chlamydia sexually transmitted infections (STIs; first catch urine via nucleic acid amplification testing, NAAT).

### Study assessments

Participants complete a baseline study visit comprised of standardized assessment via computer-assisted personal-interviewing (CAPI; including both interviewer- and self-administered questions) as well as screening for HIV/STIs. Follow-up CAPI assessments are conducted at 4, 8 and 12-month follow-ups, with repeat HIV/STI screening at the 4 and 12-month visits.

Primary Outcome: Condomless Anal or Vaginal Sex. Items assessing sexual behavior were self-administered and adapted for YTW from the AIDS Risk Behavior Assessment (ARBA) [28]. All items assessed sexual behavior in the prior 4 months. The basis for construction of the primary outcome is a set of sequential questions asking the participant to estimate the number of anal and vaginal sex partners they had in the recall period and the number of condomless sex acts by type of sex (vaginal, anal) and position (receptive, insertive) with these partners. A lead-in question asks about history of gender affirming vaginosplasty which determines deployment of questions regarding vaginal sex by position (receptive or insertive).

Mediators: Collective self-esteem, transgender adaptation and integration, HIV knowledge, motivation, behavioral skills. The collective self-esteem scale (CSES) [29] is a 16-item self-report measure that assesses a person’s thoughts and feelings regarding their social group (modified to include reference to the “transgender community”). Using a seven-point scale, participants indicated the degree to which they agree or disagree with statements regarding the reference group (1-strongly disagree to 7-strongly agree). The items form four subscales (four items each): membership (e.g., “I am a worthy member of the transgender community”); private (e.g.,“I feel good about the transgender community”); public (e.g., “In general, others respect the transgender community”); identity (e.g., “Being part of the transgender community is an important reflection of who I am”). The TG-AIM is a 15-item measure that assesses adjustment and experiences that are specific to gender identity [30]. This validated measure uses a 4-point Likert scale (0 = never, 1 = rarely, 2 = occasionally, 3 = frequently) to assess four subscales: (1) coping and gender reorientation efforts; (2) gender locus of control; (3) gender-related fears subscale; (4) psychosocial impact of gender status subscale. Although this scale has been used in previous research with transgender adults [30, 31], we have adapted this measure as appropriate for YTW.

To measure HIV knowledge, we adapted the brief HIV Knowledge Questionnaire (HIV-KQ-18) [32] for use with YTW (total 20 items). Motivation and behavioral skills are measured using items adapted from previous HIV prevention studies [33–35]. Motivation to practice safer behavior is assessed as three separate dimensions: attitudes, norms, and behavioral intentions to engage in HIV-preventive actions (total scale = 24 items). Behavioral skills for HIV prevention is assessed with 12 items that asked how “hard” or “easy” it was for participants to implement a variety of skills, including discussing sex and condom use with sexual partners, and acquiring and using condoms. Response options range from 1 = “Very Hard to Do” to 5 = “Very Easy to Do.”

### Statistical analysis

Initially, the distribution of all variables will be assessed, as will the correlations between all study variables and the primary and secondary outcomes. We will also assess for patterns of missing data. The primary anticipated reason for missing data is attrition due to loss to follow-up. We estimate 20% attrition to the 12-month follow-up assessment (375 randomized leading to 300 completers of the intervention). Outcome variables will be examined to determine which distributional models (e.g., Gaussian, Poisson) are most appropriate for subsequent statistical procedures. The primary analysis will compare differences in risk behavior at the month 4 visit between study arms using an intent-to-treat approach. Between-group differences at the month 8 and month 12 visits will similarly be compared. We will use mixed effects models with properly-chosen (based on the distribution of dependent variable) link functions to analyze longitudinal data for each study aim and examine the difference in the rate of change between intervention groups for the outcome variables. Mixed effects models explicitly model individual change across time and allow for unequal number of observations per individual (e.g., missing time points) [36]. If the 6-session Life Skills intervention does work to reduce sexual risk-taking behaviors among the sample in significantly greater magnitude than the comparison conditions, we will assess the extent to which this relationship works through several possible mediators, including increases in collective self-esteem/empowerment, transgender adaptation and integration, and HIV related information, motivation, and behavioral risk-reduction skills. For hypothesized mediators that are significantly changed by the intervention (i.e., the rate of change differed by treatment assignment), we will conduct mediation analysis to determine whether the effect of the intervention on the primary, acute outcome (i.e., at month 4 follow-up) was through the hypothesized mediator(s) [37].

### Sample size calculation

The primary power analysis is based on detecting a 40% (or greater) difference in the number of condomless sex acts between groups over the course of the study (per estimate of effect size in the pilot study) [16], expecting all groups to show improvements from baseline. Based on these data, with a power of 85% and alpha level of 0.05 and factoring in 20% attrition, we estimate needing 300 completers of the proposed intervention (LS = 120; SOC arm = 120; DN time and attention-matched control = 60).

## Discussion

We describe herein the design of the Project LifeSkills study, a randomized controlled efficacy trial of an empowerment-based, culturally-specific, and group-delivered intervention to reduce sexual risk behavior in YTW. The intervention draws both from empowerment theory and standard cognitive behavioral HIV prevention approaches to address HIV knowledge, motivation, and behavioral skills for sexual risk reduction. We believe the design of this study has several strengths, including its focus on younger transgender women; the empowerment-based approach, which is informed by the experiences of YTW themselves; and the strength of the research design.

The LifeSkills intervention is particularly innovative because it is designed to reduce sexual risk among young transgender women. No other interventions designed as randomized controlled efficacy trials and targeting transgender women are previously described in the literature. Transgender women have some of the highest rates of HIV infection in the U.S. and internationally. YTW are particularly vulnerable to sexual risk because of the intersection of sexual development and experimentation with societal marginalization and stigmatization due to emerging trans feminine identity. Thus, this study is designed to intervene early in the gender transition/gender affirmation process and to couple information about gender transition/affirmation with sexual health promotion and risk reduction. We believe that this is developmentally responsive and addresses the age range with the steepest rise in acquisition and transmission risk (i.e, ages 16-29 years).

Now in our fourth decade of the development of social and behavioral approaches to reduce HIV infection in high risk populations, prevention scientists have been slow to recognize, prioritize, and respond to transgender women’s needs. The development of the LifeSkills intervention is meant to fill this gap. It was initially developed in cooperation with YTW to reflect their experiences and needs. Given a long history of mistrust of institutions, medical providers, and scientists, the LifeSkills intervention was built from the ground up through an iterative process to merge experiential themes and prevention science via an empowerment approach [17]. The curriculum was then manualized, refined and tested in a small pre-post trial to establish proof-of-concept, feasibility, and initial efficacy [16]. Thus, the current effort is a culmination of several years of formative work and pilot testing.

Given the dearth of culturally-specific HIV prevention interventions in the published literature, this study is the first intervention to be tested in a rigorous trial of sufficient size to detect effects among YTW. It was designed to meet the standard for evidence-based interventions and to extend the base of evidence for intervention with this high-risk group. As such, if shown to be efficacious, it will be the first behavioral intervention for HIV prevention for YTW in the U.S., and, we hope, will be available for dissemination as a CDC-endorsed High Impact HIV/AIDS Prevention Project (HIP).

## Abbreviations

ARBA: AIDS Risk Behavior Assessment
CAPI: Computer Assisted Personal Interviewing
CDC: Centers for Disease Control and Prevention
CSES: Collective Self-Esteem Scale
DN: Diet and Nutrition
HIP: High Impact HIV/AIDS Prevention Project
HIV: Human Immunodeficiency Virus
IMB: Information Motivation Behavior
LS: LifeSkills
MTF: Male-to-Female
NAAT: Nucleic Acid Amplification Test
NIH: National Institutes of Health
PrEP: Pre-Exposure Prophylaxis
SOC: Standard-of-Care
STI: Sexually Transmitted Infection
YTW: Young Transgender Women

## References

[1] Baral SD, Poteat T, Stromdahl S, Wirtz AL, Guadamuz TE, Beyrer C (2013). Worldwide burden of HIV in transgender women: a systematic review and meta-analysis. Lancet Infect Dis.

[2] Herbst JH, Jacobs ED, Finlayson TJ, McKleroy VS, Neumann MS, Crepaz N (2008). Estimating HIV prevalence and risk behaviors of transgender persons in the united states: a systematic review. AIDS Behav.

[3] Schulden JD, Song B, Barrosb A, Mares-DelGrasso A, Martind CW, Ramireze R, Smith LC, Wheeler DP, Oster AM, Sullivan PS (2008). Rapid HIV testing in transgender communities by community-based organizations in three cities. Public Health Rep.

[4] Garofalo R, Deleon J, Osmer E, Doll M, Harper G (2006). Overlooked, misunderstood and at-risk: exploring the lives and HIV risk of ethnic minority male-to-female transgender youth. J Adolesc Health.

[5] Reisner SL, Vetters R, White JM, Cohen EL, LeClerc M, Zaslow S, Wolfrum S, Mimiaga MJ (2015). Laboratory-confirmed HIV and sexually transmitted infection seropositivity and risk behavior among sexually active transgender patients at an adolescent and young adult urban community health center. AIDS Care.

[6] Smith DK, Herbst JH, Zhang X, Rose CE (2015). Condom effectiveness for HIV prevention by consistency of use among men who have sex with men in the united states. Journal of acquired immune deficiency syndromes (1999).

[7] Buchbinder SP, Glidden DV, Liu AY, McMahan V, Guanira JV, Mayer KH, Goicochea P, Grant RM (2014). HIV pre-exposure prophylaxis in men who have sex with men and transgender women: a secondary analysis of a phase 3 randomised controlled efficacy trial. Lancet Infectious Disease.

[8] Grant RM, Anderson PL, McMahan V, Liu A, Amico KR, Mehrotra M, Hosek S, Mosquera C, Casapia M, Montoya O (2014). Uptake of pre-exposure prophylaxis, sexual practices, and HIV incidence in men and transgender women who have sex with men: a cohort study. Lancet Infect Dis.

[9] Grant RM, Lama JR, Anderson PL, McMahan V, Liu AY, Vargas L, Goicochea P, Casapia M, Guanira-Carranza JV, Ramirez-Cardich ME (2010). Preexposure chemoprophylaxis for HIV prevention in men who have sex with men. N Engl J Med.

[10] Kuhns LM, Reisner SL, Mimiaga MJ, Gayles T, Shelendich M, Garofalo R (2016). Correlates of PrEP indication in a multi-site cohort of young HIV-uninfected transgender women. AIDS Behav.

[11] Deutsch MB, Glidden DV, Sevelius J, Keatley J, McMahan V, Guanira J, Kallas EG, Chariyalertsak S, Grant RM (2015). HIV pre-exposure prophylaxis in transgender women: a subgroup analysis of the iPrEx trial. The lancet HIV.

[12] Smith DK, Herbst JH, Rose CE (2015). Estimating HIV protective effects of method adherence with combinations of preexposure prophylaxis and condom use among african american men who have sex with men. Sex Transm Dis.

[13] Centers for Disease Control and Prevention (CDC). Preexposure prophylaxis for the prevention of HIV infection in the united states – 2014 clinical practice guideline. Atlanta: Department of Health and Human Services; 2014.

[14] Garofalo R, Kuhns LM, Reisner SL, Mimiaga MJ (2016). Behavioral interventions to prevent HIV transmission and acquisition for transgender women: a critical review. Journal of acquired immune deficiency syndromes (1999).

[15] Leung M, Yen I, Minkler M (2004). Community based participatory research: a promising approach for increasing epidemiology’s relevance in the 21st century. Int J Epidemiol.

[16] Garofalo R, Johnson AK, Kuhns LM, Cotten C, Joseph H, Margolis A (2012). Life skills: evaluation of a theory-driven behavioral HIV prevention intervention for young transgender women. Journal of urban health : bulletin of the New York Academy of Medicine.

[17] Cotton C, Garofalo R (2016). Project life skills: developing the content of a multidimensional HIV prevention curriculum for young transgender women aged 16-24. Journal of HIV/AIDS & Social Services.

[18] Glanz K, Rimer BK, Viswanath K (2008). Health behavior and health education: theory, research, and practice.

[19] Fawcett S, Paine-Andrews A, Francisco VT (1995). Using empowerment theory in collaborative partnerships for community health and development. Am J Community Psychol.

[20] Rappaport J (1987). Terms of empowerment/exemplars of prevention: toward a theory for community psychology. Am J Community Psychol.

[21] Shearer N (2004). Relationships of contextual and relational factors to health empowerment in women. Research and theory for nursing practice.

[22] Bockting W, Kirk S (2001). Transgender and HIV: risks, prevention, and care.

[23] Fergus S (2005). Zimmerman, MA: **adolescent resilience: a framework for understanding healthy development in the face of risk**. Annu Rev Public Health.

[24] Sharpe TT, Harrison KM, Dean HD. Summary of CDC consultation to address social determinants of health for prevention of disparities in HIV/AIDS, viral hepatitis, sexually transmitted diseases, and tuberculosis. December 9-10, 2008. Public Health Rep. 2010;125(Suppl 4):11–5. 10.1177/00333549101250S404 PMC2882970 20626189

[25] Fisher JD, Fisher WA (1992). Changing AIDS risk behavior. Psychol Bull.

[26] Fisher WA, Fisher JD. A general social psychological model for changing AIDS risk behavior. In: The social psychology of HIV infection edn. Edited by Pryor JB, Reeder GD. Hillsdale: Erlbaum; 1993. p. 127-153.

[27] Metcalf C, Douglas J, Malotte C, Cross H, Dillon B, Paul S, Padilla S, Brookes L, Lindsey C, Byers R (2005). Relative efficacy of prevention counseling with rapid and standard HIV tesitng: a randomized, controlled trial (RESPECT-2). Sex Transm Dis.

[28] Donenberg GR, Emerson E, Bryant FB, Wilson H, Weber-Shifrin E (2001). Understanding AIDS-risk behavior among adolescents in psychiatric care: links to psychopathology and peer relationships. J Am Acad Child Adolesc Psychiatry.

[29] Luhtanen R, Crocker J (1992). Collective self-esteem scale: self-evaluation of one’s social identity. Personality & Social Psychology Bulletin.

[30] Sjoberg M, Walch SE, Stanny CJ (2006). Development and initial psychometric evaluation of the transgender adaptation and integration measure (TG AIM). International Journal of Transgenderism.

[31] Sanchez F, Vilain E (2009). Collective self-esteem as a coping resource for male-to-female transsexuals. J Counseling Psychology.

[32] Carey M, Schroder K (2002). Development and psychometric evaluation of the brief HIV knowledge questionnaire. AIDS Educ Prev.

[33] Amico KR, Toro-Alfonso J, Fisher JD (2005). An empirical test of the information, motivation and behavioral skills model of antiretroviral therapy adherence. AIDS Care 2005.

[34] Bryan AD, Fisher DJ, Benziger TJ (2001). Determinants of HIV risk among Indian truck drivers. Soc Sci Med.

[35] Starace F, Massa A, Amico KR (2006). Adherence to antiretroviral therapy: an empirical test of the information-motivation-behavioral skills model. Health Psychol 2006.

[36] Singer J, Willett J (2003). Applied longitudinal data analysis: modeling change and event occurrence.

[37] MacKinnon D. Introduction to statistical mediation analysis. New York: Lawrence Erlbaum Associates; 2008.

